# PI3K class II α: a novel regulator of vesicular trafficking at the base of the primary cilium

**DOI:** 10.1186/2046-2530-1-S1-P67

**Published:** 2012-11-16

**Authors:** I Franco, F Gulluni, C Costa, JP Margaria, CC Campa, E De Luca, D Monteyne, D Pérez-Morga, A Boletta, A Ranghino, GR Merlo, E Hirsch

**Affiliations:** 1 Molecular Biotechnology Center, University of Torino, Italy; 2 Massachusetts General Hospital Cancer Center, Harvard Medical School, USA; 3 Laboratoire de Parasitologie Moléculaire, Institut de Biologie et de Médecine Moléculaires (IBMM), Université Libre de Bruxelles,Belgium; 4 Dulbecco Telethon Institute, Dibit San Raffaele Scientific Institute, Italy; 5 San Giovanni Battista Hospital, University of Torino, Italy

## 

The class II phosphoinositide 3-kinase PI3K-C2α is a protein of the early endocytic compartment and the trans-Golgi network. It produces PtdIns-3-P and is characterized by a clathrin-binding site which confers to this enzyme an important role in modulating clathrin distribution and activity in the cell. However, implication of PI3K-C2α in the primary cilium biology has never been described. Through the generation of a Pik3c2a knock-out mouse strain, we discovered that PI3K-C2α was fundamental during embryonic development and that its loss conferred features of ciliopathy. In particular, homozygous mutant embryos died at midgestation and displayed laterality defects and impaired Hedgehog signaling, while heterozygous adults showed renal cysts susceptibility after kidney injury. Cilia of Pik3c2a deficient embryos were shorter and swollen and displayed a defect in accumulating Smo and Polycystin-2. In primary mouse embryonic fibroblasts, PI3K-C2α was highly enriched on vesicles at the basal body of primary cilia. In Pik3c2a-deficient cells, absence of the protein specifically caused a reduction of vesicular trafficking at the cilium base, suggesting that PI3K-C2α, through its ability to recruit clathrin and produce PtdIns-3-P, is required for the correct exchange of structural proteins and signaling molecules between the cilium compartment and the cytoplasm. Thus, our data indicate PI3K-C2α as a novel regulator of vesicular trafficking at the base of the primary cilium.

